# Draft genome sequence of fastidious pathogen *Ceratobasidium theobromae*, which causes vascular-streak dieback in *Theobroma cacao*

**DOI:** 10.1186/s40694-019-0077-6

**Published:** 2019-09-30

**Authors:** Shahin S. Ali, Asman Asman, Jonathan Shao, Amanda P. Firmansyah, Agung W. Susilo, Ade Rosmana, Peter McMahon, Muhammad Junaid, David Guest, Tee Yei Kheng, Lyndel W. Meinhardt, Bryan A. Bailey

**Affiliations:** 1 Sustainable Perennial Crops Laboratory, USDA/ARS, Beltsville Agricultural Research Center-West, Beltsville, MD 20705 USA; 2 Department of Viticulture & Enology, University of California, Davis, CA 95616 USA; 3 Department of Plant Pests and Diseases, Hasanuddin University, Jl. Perintis Kemerdekaan KM 10, Makassar, 90245 Indonesia; 4 Cocoa Research Group, Faculty of Agriculture, Hasanuddin University, Jl. Perintis Kemerdekaan KM 10, Makassar, 90245 Indonesia; 5 USDA/ARS, Northeast Area, Beltsville, MD 20705 USA; 6 Faculty of Agriculture, Muhammadiyah University of Makassar, Makassar, Sulawesi Selatan 90221 Indonesia; 7 Indonesian Coffee and Cocoa Research Institute, Jl. PB Sudirman 90, Jember, 68118 Indonesia; 8 Sydney Institute of Agriculture, School of Life and Environmental Sciences, The University of Sydney, Sydney, NSW 2006 Australia; 9 Cocoa Upstream Technology Department, Malaysian Cocoa Board, P.O. Box 30, Sg. Dulang Road, Sg. Sumun, Perak Malaysia

**Keywords:** *Ceratobasidiaceae*, Chocolate, *Rhizoctonia*, RNA-Seq, VSD

## Abstract

**Background:**

*Ceratobasidium theobromae*, a member of the *Ceratobasidiaceae* family, is the causal agent of vascular-streak dieback (VSD) of cacao, a major threat to the chocolate industry in the South-East Asia. The fastidious pathogen is very hard to isolate and maintain in pure culture, which is a major bottleneck in the study of its genetic diversity and genome.

**Result:**

This study describes for the first time, a 33.90 Mbp de novo assembled genome of a putative *C. theobromae* isolate from cacao. Ab initio gene prediction identified 9264 protein-coding genes, of which 800 are unique to *C. theobromae* when compared to *Rhizoctonia* spp., a closely related group. Transcriptome analysis using RNA isolated from 4 independent VSD symptomatic cacao stems identified 3550 transcriptionally active genes when compared to the assembled *C. theobromae* genome while transcripts for only 4 *C. theobromae* genes were detected in 2 asymptomatic stems. De novo assembly of the non-cacao associated reads from the VSD symptomatic stems uniformly produced genes with high identity to predicted genes in the *C. theobromae* genome as compared to *Rhizoctonia* spp. or genes found in Genbank. Further analysis of the predicted *C. theobromae* transcriptome was carried out identifying CAZy gene classes, KEGG-pathway associated genes, and 138 putative effector proteins.

**Conclusion:**

These findings put forth, for the first time, a predicted genome for the fastidious basidiomycete *C. theobromae* causing VSD on cacao providing a model for testing and comparison in the future. The *C. theobromae* genome predicts a pathogenesis model involving secreted effector proteins to suppress plant defense mechanisms and plant cell wall degrading enzymes.

## Background

**Fig. 1:**
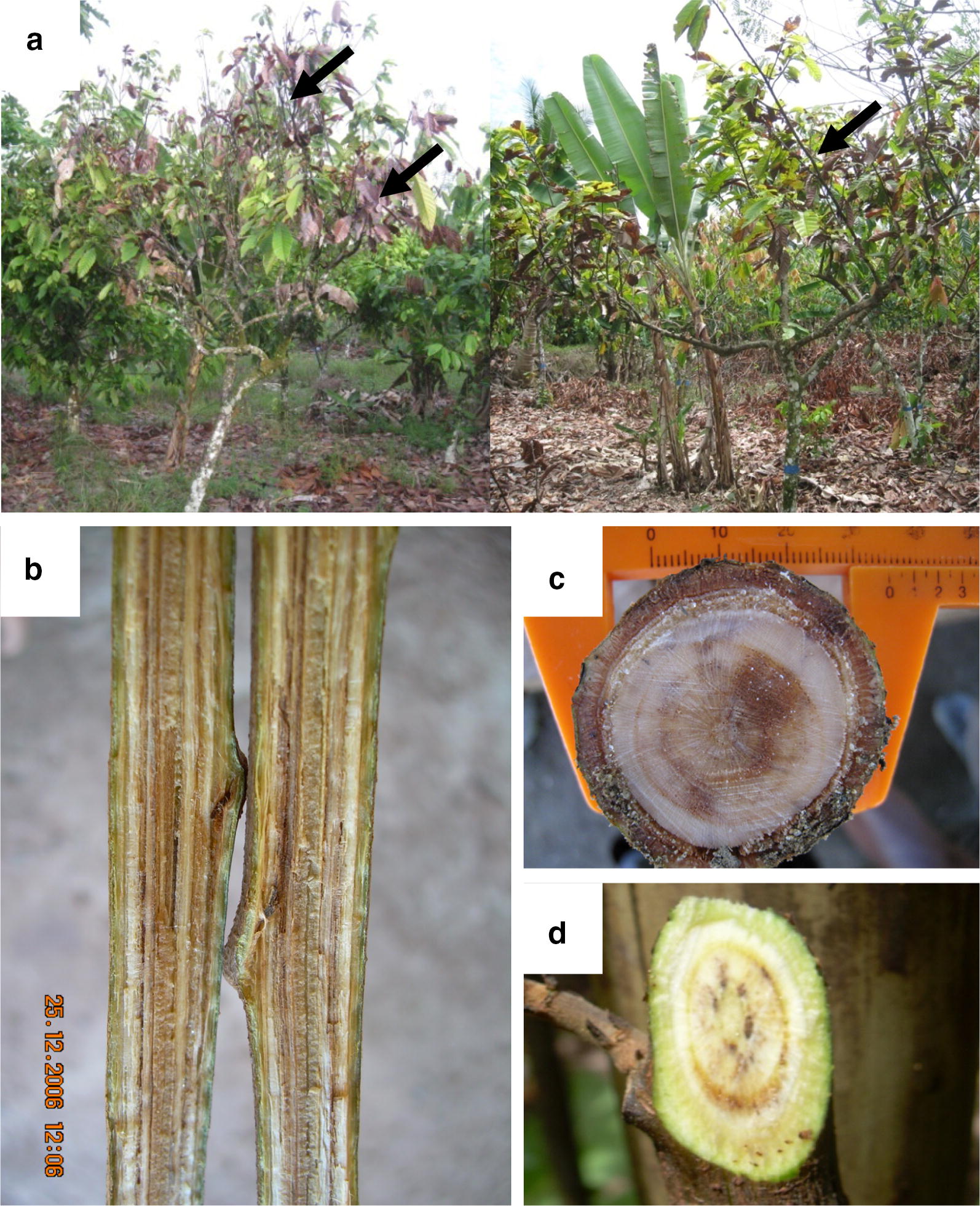
Vascular streak dieback (VSD) symptoms on cacao trees. **a** Branches killed due to VSD on mature cacao trees. **b** The visible streaks in the xylem of young stems of cacao trees. **c**, **d** Infection of transverse sections of cacao stems

*Ceratobasidium theobromae* (P.H.B. Talbot & Keane) Samuels & Keane (syn*. Oncobasidium theobromae, Thanatephorus theobromae*) is a basidiomycete fungus that causes Vascular-streak dieback (VSD), the second most important disease of *Theobroma cacao* (cacao) in Southeast Asia after *Phytophthora* pod rot (black pod) [1]. While the global loss due to black pod is around 400,000 tons annually, VSD is responsible for approximately 30,000 tons of cacao crop loss, similar to losses due to frosty pod, another disease restricted to South and Central America [1]. Established as the causal pathogen during the 1960s [2], cacao and avocado are the only known hosts of this new encounter disease [3]. The short-lived, wind-borne spores of *C. theobromae* infect the soft young leaves at the branch tip and colonize the leaf xylem. From the leaf, the hyphae reach the stem through the petiole and spread via the xylem vessels and cause vascular necrosis and dieback of the branches, sometimes killing young trees (Fig. 1). The pathogen is endemic to most cacao growing areas of Melanesia and Southeast Asia, causes severe losses locally and contributes to declining productivity and farmers abandoning cacao cultivation. Due to the limited availability of morphological and genetic diversity information and its fastidious nature, *C. theobromae* has been very difficult to study. Currently the pathogen is identified based on PCR/sequencing of ITS regions [1]. In a recent report, Oberwinkler et al. [4] suggested that the number of nuclei in the hyphal cells was inadequate as a taxonomically defining character and hence, *C. theobromae* should be renamed *Rhizoctonia theobromae*. Within the *Ceratobasidiaceae* family, *Rhizoctonia* spp. are the most widely studied due to their wide host range and global distribution. Though there are at least 5 publicly available genomes of multinucleate *R. solani* isolates with various anastomosis groups (AG1-IA, AG1-IB, AG2-2IIIB, AG3 and AG8) [5–9], there are no similar studies on binucleate *Rhizoctonia* spp. (teleomorphs: *Ceratobasidium* spp.).

The xylem-infecting pathogen grows from infected tissue onto water agar or Corticium Culture Medium (CCM) [10], but cannot be maintained in pure culture [11]. Due to its slow growth, other fungi present in the stems, petioles or leaves often overgrow *C. theobromae* during isolation attempts. When isolated in an apparent pure culture, the fungus dies out with repeated attempts at subculturing. The absence of pure culture biomass is the major constraint in obtaining good quality DNA required for molecular studies of *C. theobromae*. After multiple prolonged attempts, we obtained putative mycelia of *C. theobromae* and have carried out whole genome sequencing of the fungus for the first time. *C. theobromae* being fastidious in nature, we used the transcriptome sequence of symptomatic and asymptomatic cacao tissue to confirm the causal agent of VSD and identify its transcriptionally active genes.

## Result and discussion

**Fig. 2:**
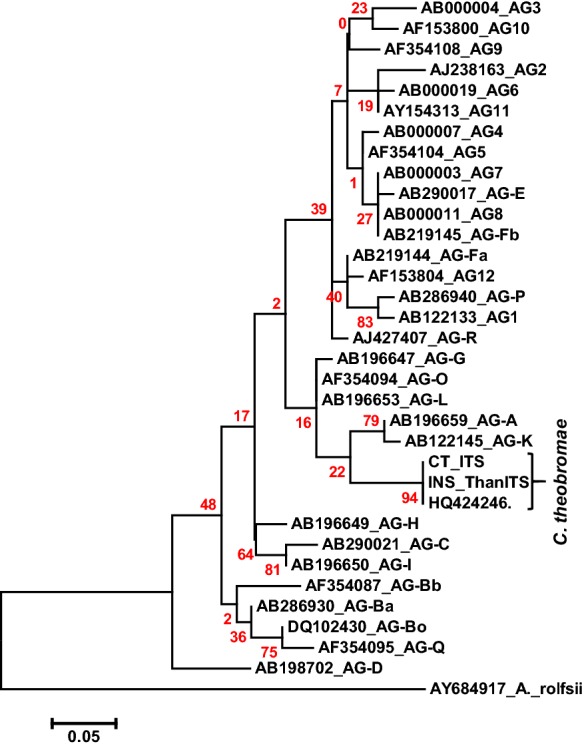
Molecular phylogenetic analysis of *Ceratobasidium theobromae* isolated from vascular streak dieback (VSD) symptomatic cacao samples from Soppeng (CT) and *in planta* strains, present in the VSD symptomatic cacao samples from Luwu District of South Sulawesi Province, Indonesia (INS), a *C. theobromae* isolate previously reported from Indonesia [11] and different *Rhizoctonia* spp. and *Ceratobasidium* spp. representing 30 anastomosis groups. The distance tree of 1000 bootstrapped data sets with the highest log likelihood (− 924.178) is shown. Branch lengths measured in the number of substitutions per site

VSD, caused by the fastidious basidiomycete *C. theobromae*, is a serious threat to the cacao industry in South East Asia [1]. To isolate the VSD causing pathogen, we collected stems and petioles showing symptoms of VSD from the Soppeng District of South Sulawesi Province, Indonesia. One isolate (CT2) from these tissues was suspected to be *C. theobromae* based on its mycelial morphology [11] on water agar, and transferred to CCM media. After 3 months of growth, mycelia were harvested and DNA extracted and subjected to ITS sequencing. BLASTn searches of the ITS sequence showed 99.85% similarity to a *C. theobromae* strain South Sulawesi 2 (GenBank: HQ424246). Strains of multinucleate *R. solani* have been assigned to 13 anastomosis groups (AG-1 to AG-13). Whereas, strains of binucleate *Rhizoctonia* spp. with *Ceratobasidium* teleomorphs have been grouped into 21 anastomosis groups designated as AG-A to AG-U [12]. Publicly available ITS sequences were obtained from GenBank for different *Rhizoctonia* spp. and *Ceratobasidium* spp. representing 30 of the anastomosis groups mentioned above. A phylogenetic study involving the ITS sequence of the *C. theobromae* isolate CT2 along with isolate previously reported from Indonesia and the 30 anastomosis groups shows that *C. theobromae* forms a separate clade and the closest related anastomosis groups are AG-A and AG-K (Fig. 2). Similar results were also reported by Samuels et al. [11] who also confirmed the binucleate nuclear condition of *C. theobromae* mycelia and observed hyphal anastomoses. Using a nested PCR assay, 7 VSD symptomatic cacao stem samples from the Luwu District of South Sulawesi Province, Indonesia were confirmed to be infected by *C. theobromae*, and alignment of the ITS sequences from the 7 samples showed no variation (result not shown). There was one base pair difference between the ITS sequences of CT2 and the set of samples from Luwu (result not shown), indicating possible regional variation among the *C. theobromae* strains. Among the confirmed *C. theobromae* infected VSD symptomatic stem samples, 4 were subjected to RNA-Seq analysis along with 2 asymptomatic control samples.

The initial genome assembly obtained was 44,646,720 bp and consisted of 105,861 contigs with a N50 length of 36,882 bp. Though the average GC content of the assembly was 49.25%, many of the smaller contigs had GC content greater than 60%, indicating the presence of bacterial DNA in the assembly, though the mycelial culture in liquid CCM appeared clean. Although the bacterial contamination may have been random, another possibility is that the bacterial contaminant may have synergistic effects on the growth of *C. theobromae* possibly being necessary for its growth in vitro and/or contributing to the pathogens fastidious nature. A recent report on the presence of a bacterial endosymbionts in *R. solani* AG 2-2IIIB and its role in virulence [13], makes it relevant to resolve the presence of the bacterial genome along with *C. theobromae.* To remove the bacterial contamination from the genome assembly we combined two post-assembly cleaning approaches. A metagenome binning approach was used to filter out the fungal and bacterial genome. The second approach was based on GC-content and multiple similarity searches to detect potential contaminating sequences in the initial assembly. These post-assembly cleaning procedures have some advantages over pre-assembly cleanup [14].

**Fig. 3:**
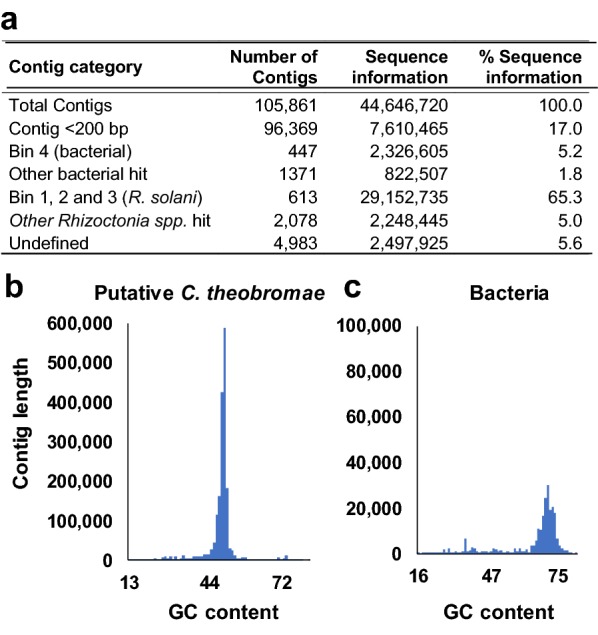
Detection of bacterial contaminant in the initial genome assembly of *Ceratobasidium theobromae*. **a** The percentage of smaller contigs (< 200 bp), contigs sorted by MetaBAT2 and bacterial/*Rhizoctonia* blast hits over the contigs. **b**, **c** Illustrate the different distribution of GC contents in the sequences considered as bacterial and putative *C. theobromae*

Engaging MetaBAT2, an automated metagenome binning software tool to reconstruct single genomes [15], 105,861 contigs were grouped into 4 genome bins (Table 1). Bin 1 included 28.29 Mbp, with an average GC content of 49.33% and the closest representation was *R. solani*. Bin 2 and 3 were also represented as *R. solani* and the total sequence included was small (Table 1). Altogether, 613 contigs from the three bins correspond to 29.15 Mbp of sequence information and was considered as putative *C. theobromae* genome sequence. The fourth bin included 2.33 Mbp, with an average GC content of 72.25% and the closest representation was *Actinomyces* sp. The bacterial genome showed 83.5% BUSCO [16] completeness (Table 1). Further studies are needed to see if the same bacterium is always associated with *C. theobromae* and has any role in its virulence or fastidious nature. MetaBAT2 was unable to bin 104,801 contigs, which were manually processed based on GC-content and multiple similarity searches. For that, we first eliminated 96,369 smaller contigs (< 200 bp), comprising 7.6 Mbp sequence information as possible bacterial contamination or bad sequence (Fig. 3a). The remaining contigs were subjected to BLASTn searches against the NCBI nucleotide (nt) database (version December, 2018) and a closely related *R. solani* genome (GenBank: JATN00000000) with an *e*-value cutoff of 1e−5. In total, 1371 contigs corresponding to 822.5 Kbp of sequence information were identified as bacterial in origin based on BLASTn hits to bacterial genes or genomes. A BLASTn search against the *Rhizoctonia* spp. identified 2078 contigs corresponding to 2.25 Mbp of sequence information and were considered as additional putative *C. theobromae* genome sequence. Another 4983 contigs (corresponding to 2.49 Mbp) could not be attributed to either bacteria or *Rhizoctonia* spp. (Fig. 3a). As a benefit of the doubt, we considered those to be part of the *C. theobromae* genome. Comparison of GC content and contig length of the predicted bacterial and *C. theobromae* contigs show clear difference (Fig. 3b, c). Using BBMap version 37.58 [17], 333,747,598 short reads (92.65% of the initial reads) were aligned to the 7674 contigs considered to be of *C. theobromae* genome. These reads were re-assembled using SPAdes and resulted in a 33.90 Mbp genome sequence with approximately 930× coverage.

**Table 1:** Clustering of the initial 105,861 genome assembly contigs (44.64 Mbp) into genome bins using MetaBAT2

Genome bins	Bin 1	Bin 2	Bin 3	Bin 4
Closest representation^a^	*Rhizoctonia solani*	*R. solani*	*R. solani*	*Actinomyces* sp.
Total contig length (bp)	28,287,668	496,199	368,868	2,326,605
Total contig number	600	5	8	447
BUSCO Completeness (%)	93.4	NA	NA	83.5
Max Contig size (bp)	589,277	263,186	109,092	30,075
Min Contig size (bp)	2653	25,328	9627	1509
Mean Contig size (bp)	47,146	99,240	46,109	5205
N50 Contig length (bp)	93,535	263,186	81,760	6567
Mean GC content	49.33%	49.23%	50.41%	72.25%
Max GC content	54.13%	49.61%	51.48%	79.16%
Min GC content	44.57%	47.88%	49.44%	64.56%

^a^Based on BLASTx search of the contigs against Nr-database

The estimated *C. theobromae* genome is smaller than closely related Ceratobasidiales like *R. solani* strains (56.02–36.9 Mbp) [5–9] and *Botryobasidium botryosum* (45.75 Mb) [18]. The assembly consisted of 6878 contigs with N50 length of 70,517 bp (Table 2). The overall GC content of the *C. theobromae* genome is 44.81%, which is close to different strains of *R. solani* (43.8–48.4%) [5–9], the only other sequenced species in the *Ceratobasidiaceae* family. Among the assembled contigs, Contigs 1191 and 8134 were identified to be part of the mtDNA and carry the ITS and 28S ribosomal RNA gene sequence of *C. theobromae*. The ITS sequence acquired was identical to that obtained by PCR from the DNA submitted for sequencing and the 28S ribosomal RNA sequence was identical to that submitted by Samuels et al. [11] for *C. theobromae.* For quantitative assessment of genome completeness, BUSCO [16] analysis was conducted that indicated *C. theobromae* contains 99.1% of examined loci (85.3% complete genes and 13.3% fragmented genes). The ab initio gene prediction generated 9264 protein-coding genes in the *C. theobromae* genome with an average sequence length of 2,168 bp (Table 2). In comparison to the five strains of *R. solani,* the assembled CT2 genome has between 1225 to 4156 less genes.

**Table 2:** Genome assembly and annotation statistics of *Ceratobasidium theobromae*

	*C. theobromae*
Total Contig length (bp)	33,899,105
Contig numbers	6878
BUSCO completeness (%)	99.1%
GC content	44.81%
N50 Contig length (bp)	70,517
Max Contig size (bp)	589,277
Min Contig size (bp)	200
Mean Contig size (bp)	4930
Gene number	9264
Total gene length (bp)	20,074,964
Average gene length (bp)	2168.07
Gene density^a^	0.592
Number of expressed genes^b^	3550
Genes with GO annotation^c^	5364
Genes within KEGG pathway	3055

^a^CDS bases/total genome bases

^b^Only gene models with ≥ 10 raw reads, detected in any of the infected plant samples

^c^Gene models with E < 10^−5^ for BLASTn against Uniport Gene Ontology database

Functional annotation of the 9264 predicted genes showed that 5364 (58%) could be assigned GO terms and 3055 (33%) could be mapped to the KEGG pathway database (Table 2; Additional file 1: Sheet 1). KEGG pathway analysis of the whole *C. theobromae* genome and two publicly available *R. solani* genomes (AG1-IA: AFRT00000000 and AG3: JATN00000000) showed very similar results, with nearly complete major metabolic pathways for both the species (Additional file 1: Sheet 3). This is another indication that, though the predicted *C. theobromae* genome is smaller with fewer genes compared to *R. solani*, its gene complement is almost complete. Bidirectional BLASTp analysis conducted with *Rhizoctonia* spp. has identified 800 species-specific genes for *C. theobromae* (Additional file 1: Sheet 2). Though the major part of these species-specific genes encodes hypothetical proteins, the most relevant difference was the 11 *C. theobromae* specific putative effector proteins (Additional file 1: Sheet 2).

*C. theobromae* being fastidious in nature, Koch’s postulates are not possible; therefore, we adopted the concept of sequence-based identification of the microbial pathogen [19] and relied on the transcriptome sequence of symptomatic and asymptomatic cacao tissue. RNA-Seq analysis of four VSD stem segments verified to carry the *C. theobromae* ITS sequence identified 3551 transcriptionally active genes (with ≥ 10 raw reads) from the *C. theobromae* transcriptome, compared to just four *C. theobromae* genes showing any read detection in the two asymptomatic samples (Additional file 1: Sheet 1). The relatively low level detection of *C. theobromae* transcripts in the VSD symptomatic tissue may be due to the low titer of the fungal RNA in the samples. This is consistent with earlier reports that the fungal load of *C. theobromae* in the symptomatic tissues is very low and the same PCR-based detection techniques used here often fail in VSD symptomatic cacao tissues [11].

Self-assembly of non-cacao RNA-Seq reads from the 4 VSD symptomatic tissue samples incorporated reads ranging from 88,978 to 746,046 (Additional file 2: Sheet 1–4), depending on the sample, representing less than 0.2% of the total reads of those samples. The number of de novo assembled transcripts from the four samples ranged from 425 to 2271 (Additional file 2: Sheet 1–4). When compared to sequences in Genbank, most of the de novo assembled transcripts showed homology to the related *R. solani* AG3. Almost every transcript showed higher homology to the *C. theobromae* genes predicted from the genome assembled in this study compared to *R. solani* AG3 genes (Additional file 2). A closer inspection of the alignments between the self-assembled transcripts and the predicted genes indicates that the self-assembled transcripts are much shorter and have gaps compared to predicted genes, but otherwise have near perfect sequence matches (result not shown). This further strengthens our assertion that we have sequenced the genome of the fungus causing VSD in cacao and that the predicted genes from the assembled genome are nearly complete.

After aligning the *C. theobromae* specific RNA-Seq reads (Table 3) from the 4 VSD symptomatic tissue samples against its genes assembled in this study, variant calling generated 4326 putative single nucleotide polymorphisms (SNPs) and small insertions and deletions (INDELs). After filtering the variants (SNPs and INDELs) as mentioned in the methods, a total of 2278 putative CDS (coding DNA sequence)-based variants were obtained in 1051 *C. theobromae* genes (Additional file 2: Sheet 5). Among the 4 libraries, the total number of putative variants (QUAL ≥ 999, DP ≥ 30 and GQ ≥ 40) ranged between 307 and 1673 (Table 3). The ratios between heterozygous and homozygous variants ranged between 0.021 and 0.063 (Table 3). Though the sampling size is small and these variants are not validated yet, the presence of very low heterozygosity suggests local genetic variability among *C. theobromae* isolates from the South Sulawesi Province of Indonesia may be limited compared to regional variability. Moreover, there was not a single homozygous and heterozygous variant combinations for the same allele in the data set (Additional file 2: Sheet 5) suggesting, at a minimum, the pathogen is homothallic. Although sexual reproduction is known to occur in *C. theobromae* [11], we failed to identify mating type genes within the *C. theobromae* genome.

**Table 3:** List of filtered transcriptome sequence variants in RNA-Seq libraries from VSD-infected stems

RNA-Seq library	No. of RNA reads in each library	No. of variants (QUAL ≥ 999, DP ≥ 30, and GQ ≥ 40)			
		Homozygous alternate		Heterozygous	
		SNPs	INDELs	SNPs	INDELs
INS_19	108,224	258	39	10	0
INS_23	283,192	620	135	26	0
INS_31	888,258	1357	280	34	2
INS_57	109,242	279	37	16	4

Proteins secreted by plant pathogenic fungi have the potential to interact with and alter host cells and therefore, their identification and characterization is essential to understanding virulence and the mechanism of infection [20]. As predicted by SignalP version 5.1 [21], 998 proteins were considered potential components of the *C. theobromae* secretome (Additional file 1: Sheet 1), accounting for 10.7% of its proteome. Fungal effectors among the secretome were identified using the machine learning program EffectorP 2.0 [22]. The 138 effector proteins predicted (Additional file 1: Sheet 4) are considered of importance due to their potential to suppress the pathogen-associated molecular pattern triggered plant immunity and are potential candidate genes for future studies on virulence, symptom expression and host range. Although most of these effector proteins have homologs in the *R. solani* genomes, most were identified as hypothetical proteins. The predicted secreted proteins and effectors in five *R. solani* strains range between 391–1142 and 68–126 respectively [7].

To understand the potential *C. theobromae* genes involved in the modification and degradation of cell wall and other organic matter, the predicted genes were analyzed by BLASTp against the Carbohydrate-Active enzymes database (CAZymes) using a threshold value of E < 10^−10^ and > 40% sequence identity. A total of 119 CAZymes families mapping to 664 predicted *C. theobromae* proteins were identified (Table 4). The web server based automated CAZyme annotation using dbCAN2 [23] also generated similar result and identified 663 *C. theobromae* proteins as potential CAZymes. Among the potential CAZymes, 355 are predicted to encode secreted proteins based on the presence of a signal peptide (Table 4; Additional file 1: Sheet 1). Glycoside hydrolases (GH) formed the largest group followed by glycosyltransferases (GT) and auxiliary activities (AA) (Table 4). Among the secreted CAZymes, again the largest group is GH followed by AA and polysaccharide lyases (PL). Amongst these large families, PL1, 3, GH28, GH43 and CE8 are often involved in pectin degradation, a component of the plant primary cell wall and middle lamella [24]. Other enzymes (AA9, GH5, GH3, and GH16 for example) often target cellulose and hemicellulose [25]. Together, the genes encoding secreted CAZymes targeting pectin, cellulose, hemicellulose and xylan during infection are expected to support the infection and colonization of cacao by *C. theobromae* (Additional file 1: Sheet 1). In addition to secreted effectors and plant cell degrading enzymes, fungi have a broader arsenal of secreted proteins/enzymes at their disposal when causing disease. Among the non-CAZyme and non-effector secreted protein coding genes, proteases/peptidases are the largest group with known putative functions, followed by lipases and Cytochrome P450 genes (Additional file 1: Sheet 1). However unidentified hypothetical proteins remain the largest group and require more detailed study.

**Table 4:** Number of CAZymes family genes of *Ceratobasidium theobromae*

CAZymes family^a^	Total genes	Secreted^b^	Non-secreted^b^
AA3	17	5	12
AA1	15	13	2
AA5	12	8	4
AA9	22	22	0
Other AA (5)	14	8	6
CBM1	15	7	8
CBM13	24	15	9
CBM5	11	7	4
Other CBM (8)	22	12	10
CE4	18	14	4
CE16	13	5	8
CE8	11	10	1
Other CE (6)	17	11	6
GH0	13	7	6
GH43	15	7	8
GH13	13	6	7
GH3	12	6	6
GH28	19	17	2
GH5	24	13	11
GH16	30	16	14
GH18	19	7	12
GH7	12	10	2
Other GH (44)	134	71	63
GT2	15	0	15
GT4	15	0	15
Other GTs (28)	70	5	65
PL1	26	23	3
PL3_2	19	17	2
Other PLs (5)	16	11	5

^a^Number within parentheses indicates the number of CAZymes families

^b^As determined by SignalP, version 5.1 and BLASTp search against Carbohydrate-Active enzymes database at the threshold value of E < 10^−10^ and > 40% similarity

## Conclusions

The assembled *C. theobromae* genome and its analysis provide insight into the genetic makeup of this important cacao pathogen. *C. theobromae* has the tools within its relatively compact 33.89 Mbp genome, like other plant pathogens, to cause plant disease. The *C. theobromae* genome presented supports a typical pathogenesis model, where the fungus secrets effector proteins involved in plant defense suppression along with enzymes required for degradation of cell walls and other cell components. Why the pathogen should be fastidious is unclear and whether its association with *Actinomyces* sp. has anything to do with the virulence or fastidious nature, requires more study. Although, a preliminary variant study suggests limited regional variation, future exploitation of the assembled genome should provide the basis for a better understanding of the genetic variability of the pathogen and support the development of potential disease resistance sources. The effector proteins identified here-in offer potential targets for molecular manipulation and resistance development. Due to the high number of genes that encode hypothetical proteins in this genome and limited detection of *C. theobromae* transcripts *in planta*, more research is needed to fully understand the disease mechanism of this fungus.

## Materials and methods

### Isolation and DNA extraction

VSD symptomatic cacao petioles were collected from Soppeng District of South Sulawesi Province, Indonesia. Petioles were surface sterilized by submerging in 6% (v/v) sodium hypochlorite for three minutes followed by three rinses in sterile water. Segments were cut transversely exposing the xylem tissue and placed on water agar. Slow growing mycelium was transferred to liquid CCM [10] then transferred to fresh liquid CCM a second time. After a total of 3 months in liquid CCM at 25 °C, mycelia were harvested in 1.5 ml microtubes and collected by centrifugation (14,000 rpm for 15 min). The mycelial pellet was freeze dried and shipped to USDA-APHIS-PPQ facility in Beltsville, USA and transferred to the USDA-ARS Sustainable Perennial Crops Laboratory in Beltsville after inspection. DNA was extracted from the mycelia as described by Ali et al. [26].

### Isolation of RNA from symptomatic plant material

VSD symptomatic and asymptomatic cacao stems were collected from the Luwu District of South Sulawesi Province, Indonesia and cut into 10 cm segments. Samples were freeze dried and shipped as mentioned above. Freeze dried cacao stems were ground in a mortar and pestle in liquid nitrogen and RNA was extracted as described by Bailey et al. [27].

### Molecular identification of *C. theobromae*

For species confirmation of the mycelial DNA samples, PCR amplification of the ITS region of the template DNA of 5 *C. theobromae* samples, all originating from the same initial water agar culture, were performed using the ITS4 and ITS5 primers described by White et al. [28]. PCR amplification, product purification and sequencing was performed as previously described by Ali et al. [26]. Similarly, to confirm the presence and absence of *C. theobromae* in the symptomatic and asymptomatic cacao stems, cDNA was synthesized from the extracted RNA as described by Bailey et al. [27] and diluted cDNA (1:20) was subjected to nested PCR assays, first using fungal specific ITS 4 and 5 primers [28] followed by *C. theobromae* specific primers Than_ITS1 and 2 [11]. PCR products were sequenced as mentioned above.

Molecular phylogenetic analysis of *C. theobromae* isolates and different *Rhizoctonia* spp. representing 30 anastomosis groups [29] was carried out based on DNA sequence data of ITS 1 and 2 regions. Sequences were aligned using the ClustalW2 tool [30] under default settings. A phylogenetic tree was reconstructed using the Maximum Likelihood method based on the Poisson correction model [31] in MEGA6 [32].

### Genome sequencing, assembly and binning

*Ceratobasidium theobromae* isolate CT2 genomic DNA was sequenced using Illumina paired-end short-read technology (library preparation and sequencing done by Beijing Genome Institute, Shenzhen, China). For the initial assembly, 360,198,964 short reads (100 bp) were trimmed using BBMap version 37.58 [17] and were assembled using SPAdes Genome Assembler version 3.11.0 [33] in read error correction and assembling mood. K-mers was set at K21, K33, K55. Genome bins were recovered based on tetranucleotide frequencies and read coverage using MetaBAT2 [15] with default parameters.

### Ab initio gene prediction

The ab initio gene prediction was performed from the assembly results using AUGUSTUS version 2.7 [34] trained with *R. solani* AG3 gene models (GenBank: JATN00000000). The predicted proteins were compared against NCBI non-redundant (NR) protein databases by BLASTp to identify biological functions [35]. Open reading frames were also annotated using Blast2GO (http://www.blast2go.com/b2ghome) [36] and the KEGG–database of metabolic pathways [37].

### Transcriptome sequencing

To validate the expression of the *C. theobromae* predicted genes, RNA-Seq was performed on RNA from 4 independent field collected VSD symptomatic cacao stems (validated as carrying *C. theobromae* above). Two asymptomatic samples were also included. RNA-Seq library preparation and paired-end sequencing was performed using the Illumina HiSeqX platform by Beijing Genome Institute, Shenzhen, China. RNA reads from RNA-Seq libraries ranging from 72 to 104 million reads in fastq format were trimmed up using BBDuk version 37.58 [17], using adapters.fa with parameters ktrrim = r, k = 23, mink = 11, hist = 1, tpe, tbo. Trimmed reads were aligned using HISAT2 2.1.0 [38] to the CDS of the *C. theobromae* genome assembled in this study.

### Mining of putative variants

RNA-Seq reads in fastq format from each VSD symptomatic library (INS_19, INS_23, INS_31 and INS_57) were aligned to the CDS of the *C. theobromae* genome assembled in this study using a memory-efficient short-read aligner Bowtie-0.12.7 [39] and the variant calling and quality control was performed as previously described by Ali et al. [40]. In short, the files were outputted into SAM and converted to BAM format using SAMtools [41]. Variant calling was performed using SAMtools mpileup and bcftools with default parameters. The resulting VCF file was filtered manually using Excel functions. All variants (SNPs and INDELs) with QUAL < 999 and DP < 30 were removed and further filtered based on genotype quality (GQ) and retained if there was at least one library supporting it (GQ ≥ 40) (Additional file 2: Sheet 5).

### De novo transcriptome assembly

To further validate the assembled genome of *C. theobromae*, the causal agent of VSD, we conducted independent de novo transcriptome assemblies of the non-cacao reads from the 4-field collected VSD symptomatic cacao stems as validated above. Trimmed reads for each sample were initially aligned using HISAT2 2.1.0 [38] to the CDS of the cacao genome [42], and the unaligned reads were assembled using SPAdes Genome Assembler version 3.11.0 [33] with K-mers set at K23. The ab initio gene prediction was performed from the assemblies using AUGUSTUS version 2.7 [34] trained with *R. solani* AG3 gene models. The predicted transcriptomes were compared against *C. theobromae* genome assembled in this study and closely related *R. solani* genome (GenBank: JATN00000000) by BLASTn and against NCBI non-redundant (NR) protein databases by BLASTx analysis.

## Supplementary information

**Additional file 1.** *C. theobromae* genome and predicted genes.

**Additional file 2.** *C. theobromae* de novo transcriptome assemblies and variants.

## Data Availability

The complete nucleotide sequence assemblies and the Whole Genome Assembly of the *C. theobromae* isolate CT2 has been deposited at GenBank under the Accession SSOP00000000, under BioProject PRJNA524910. The combined transcriptome assembly from multiple tissues have been uploaded as additional files.

## Abbreviations

AA: auxiliary activities
AG: anastomosis group
BLAST: basic local alignment search tool
bp: base pair
BUSCO: benchmarking universal single-copy orthologs
CAZyme: carbohydrate-active enzyme
CCM: corticium culture medium
CDS: coding DNA sequence
CE: carbohydrate esterase
DP: raw read depth
GC: guanine–cytosine
GH: glycoside hydrolases
GO: gene ontology
GQ: genotype quality
GT: glycosyltransferases
INDEL: small insertions and deletions
ITS: internal transcribed spacer
Kbp: kilobase pair
KEGG: kyoto encyclopedia of genes and genomes
Mbp: megabase pair
NCBI: National Center for Biotechnology Information
PCR: polymerase chain reaction
PL: polysaccharide lyases
RNA-Seq: ribonucleic acid sequencing
SNP: single nucleotide polymorphism
VSD: vascular-streak dieback
